# CA-125 and CCL2 may indicate inflammation in peritoneal dialysis patients

**DOI:** 10.1590/2175-8239-JBN-2020-0255

**Published:** 2021-05-24

**Authors:** Wander Valadares de Oliveira, Sylvia Dias Turani, Maria Aparecida Silva Marinho, Sérgio Wyton Lima Pinto, Alba Otoni, Roberta Carvalho Figueiredo, Danyelle Romana Alves Rios

**Affiliations:** 1 Universidade Federal de São João Del Rei, Campus Centro Oeste, São João Del Rei, MG, Brasil.; 2 Complexo de Saúde São João de Deus, Centro de Nefrologia, Divinópolis, MG, Brasil.

**Keywords:** Peritoneal Dialysis, CA-125 Antigen, Chemokine CCL2, Chronic Kidney Disease, Inflammation, Diálise Peritoneal, Antígeno Ca-125, Quimiocina CCL2, Doença Renal Crônica, Inflamação

## Abstract

**Introduction:**

Progressive structural changes in the peritoneal membrane occur over the course of treatment in peritoneal dialysis (PD), resulting in an increase in cytokines such as CCL2 and structural changes in peritoneal membrane triggering an increase in CA-125 in dialysate, which reflects a probable local inflammatory process, with possible loss of mesothelial cells. Thus, the current study aimed to evaluate the association between plasma and CCL2 and CA-125 dialysate levels in patients undergoing PD.

**Methods:**

Cross-sectional study was conducted with 41 patients undergoing PD. The assessments of CA-125 and CCL2 levels were performed using a capture ELISA. Correlations were estimated using Spearman's correlation and the investigation of the association between the explanatory variables (CCL2) and response variable (CA-125) was done for crude ratio of arithmetic means and adjusted utilizing generalized linear models.

**Results:**

A moderate positive correlation was observed between the levels of CA-125 and CCL2 in the dialysate (rho = 0.696). A statistically significant association was found between the levels in the CCL2 and CA-125 dialysate (RoM=1.31; CI = 1.20-1.43), which remained after adjustment for age (RoM = 1.31; CI=1.19-1.44) and for time in months of PD (RoM=1.34, CI=1.22-1.48).

**Conclusion:**

The association of CA-125 levels with CCL2 in the dialysate may indicate that the local inflammatory process leads to temporary or definitive changes in peritoneal membrane. A better understanding of this pathogenesis could contribute to the discovery of new inflammatory biomarkers.

## Resumo

**Introdução:**

Alterações estruturais progressivas na membrana peritoneal ocorrem no decorrer do tratamento em diálise peritoneal (DP), resultando em um aumento de citocinas como CCL2 e alterações estruturais na membrana peritoneal desencadeando um aumento de CA-125 no dialisato, o que reflete um provável processo inflamatório local, com possível perda de células mesoteliais. Assim, o presente estudo teve como objetivo avaliar a associação entre CCL2 e CA-125 no plasma e no dialisato de pacientes submetidos à DP.

**Métodos:**

Foi realizado um estudo transversal com 41 pacientes submetidos à DP. As avaliações dos níveis de CA-125 e CCL2 foram realizadas utilizando ELISA de captura. As correlações foram estimadas usando a correlação de Spearman, e a investigação da associação entre as variáveis explicativas (CCL2) e a variável resposta (CA-125) foi feita pela razão bruta das médias aritméticas e ajustada utilizando modelos lineares generalizados.

**Resultados:**

Foi observada uma correlação positiva moderada entre os níveis de CA-125 e CCL2 no dialisato (rho = 0,696). Foi encontrada uma associação estatisticamente significativa entre os níveis no dialisato de CCL2 e CA-125 (RoM=1,31; IC = 1,20-1,43), que permaneceu após ajuste por idade (RoM = 1,31; IC=1,19-1,44) e pelo tempo de DP em meses (RoM=1,34, IC=1,22-1,48).

**Conclusão:**

A associação dos níveis de CA-125 com CCL2 no dialisato pode indicar que o processo inflamatório local leva a alterações temporárias ou definitivas na membrana peritoneal. Uma melhor compreensão desta patogênese pode contribuir para a descoberta de novos biomarcadores inflamatórios.

## Introduction

Currently in Brazil, about 133,000 patients with chronic kidney disease (CKD) are undergoing dialysis, of which about 92.3% undergo hemodialysis and 7.7% peritoneal dialysis (PD)^1^^,^ 2.

The success of PD is totally dependent on a healthy and functional peritoneal membrane^3^. However, progressive structural changes in peritoneal membrane occur in PD, including loss of mesothelial cells, expansion of the submesothelial extracellular matrix, and neoangiogenesis^4^^,^ 5. The increase in permeability and, consequently, impairment or loss of the function of solute transport and ultrafiltration of peritoneal membrane promotes the negative clinical outcome and may change with time of treatment. Solute transport and ultrafiltration are the most important aspects of peritoneal function and should be monitored, longitudinally, in patients undergoing PD. One of the main strategies for estimating peritoneal membrane functions is the peritoneal balance test (PET), which assesses the rate of solute transport by peritoneal membrane. However, the PET result fails to reflect the integrity of the peritoneal membrane, that is, the loss of effective mesothelial cells from the peritoneum. In addition, analytical changes, inherent to the methodology for performing PET, such as colorimetry for example, may overestimate the value of creatinine in dialysate when glucose levels are increased^6^^,^ 7. Because of this, the search for new molecular biomarkers potentially representative of peritoneal morphofunctional changes becomes increasingly imminent.

The Cancer Antigen 125 (CA-125) in serum is traditionally known as a tumor marker in ovarian cancer. Because it is also produced by the mesothelial cells of the peritoneum and is unable to overcome the pores of the peritoneal membrane due to its high molecular weight (>200 kD), CA-125 has been proposed as an alternative for assessing the integrity of peritoneal membrane^8^^,^ 9. A cohort study developed by Otoni *et al.* (2000)^10^, with 15 patients at the beginning of PD, showed a significant reduction in the levels of CA-125 in the dialysate after about 60 days of follow-up (22.0 U/mL ± 4.5 vs 4.8 U/mL ± 1.3). The increase in CA-125 in the dialysate may occur mainly due to the intraperitoneal inflammatory process that stimulates the degradation of mesothelial cells in peritoneal membrane^11^^,^ 12.

This inflammatory process is characterized by increased levels of cytokines, such as interleukin (IL) 2, IL-6, IL-10, IL-17, and chemokines, such as the monocyte-1 chemo-attractive protein (MCP-1), also known as CCL2 (C-C *Motif Chemokine Ligand* 2)^13^^,^ 14. Studies have shown that CCL2 has an important role in the pathogenesis of several inflammatory and fibrosis diseases, including diabetic nephropathy^15^, renal fibrosis^16^, and even peritoneal fibrosis^17^. Based on these statements, high levels of CCL2 in the dialysate would indicate the presence of a local inflammatory process. CCL2 would act as a mediator for the recruitment and activation of monocytes/macrophages, which are well known for releasing pro-fibrotic cytokines, such as the transforming growth factor (TGF-β) and the fibroblast growth factor, which in turn promote the epithelial-mesenchymal transition, loss of mesothelial cells, increased levels of CA-125, and over time, loss of peritoneal membrane integrity^18^.

Although PD is not yet the renal replacement therapy of choice for most institutions that offer this type of service in Brazil and worldwide, this type of dialysis offers a safe, and sometimes the only alternative to performing renal replacement therapy and maintaining people with terminal CKD alive. Thus, finding new strategies that can contribute to the improvement of the technique guaranteeing a higher survival of the PD patient are fundamental to leverage adherence to this type of renal replacement therapy. However, studies on the integrity and functionality of peritoneal membrane, which determine the permanence and management of patients on PD, are still scarce in the literature. In this sense, this study aimed to assess the association between CCL2 plasma and dialysate levels and CA-125 dialysate levels in patients undergoing PD.

## Material and Methods

### Study design

This was a cross-sectional study.

### Study population

#### Inclusion Criteria

Patients on PD for at least 90 days and aged 18 years or older were recruited at the Nephrology Center of the São João de Deus Health Complex - Divinópolis/MG, Brazil in August 2011.

### Exclusion criteria

Of the total 74 eligible patients, 33 (44%) patients were excluded for meeting any of these conditions: the presence of acute diseases, autoimmune diseases, neoplasms, being HIV-positive, having had an episode of peritonitis a month before and/or one month after the evaluation, pregnancy, and being unable to sign the informed consent form.

### Study protocol

Data collection was carried out in August 2011 during consultations held monthly by patients at the Nephrology Center of the São João de Deus Health Complex. Biological samples were collected, and information on health conditions and medication use were obtained from the patients’ medical records.

All participants had 5 mL of venous blood collected using polyethylene syringes and transferred to tubes containing the anticoagulant ethylenediaminetetraacetic acid (EDTA). The samples were centrifuged at 3,500 rpm at room temperature for 15 minutes in a Novatecnica^®^ model NT815 centrifuge to obtain the plasma. The plasma was aliquoted in Eppendorf^®^ tubes and stored at -80ºC until the moment of the measurements. About 10 mL of dialysate was also collected through drainage, by force of gravity, from the dialysis bath, during time 0 of the PET, using a sterile bottle. These samples were later aliquoted in Eppendorf^®^ tubes and stored at -80ºC until the moment of the dosages.

### Study variables

#### Response variable

Levels of CA-125 (U/mL) in dialysate and plasma and dialysate CCL2 levels (pg/mL)

The determination of CA-125 in dialysate and CCL2 levels in plasma and dialysate was performed using the kit *Quantikine Human* CA-125/MUC16 and CCL2 (R & D Systems, Minneapolis, USA) capture ELISA, strictly following the manufacturer’s instructions. The reactions were read using the microplate reader VersaMax *Microplate Reader* - MOLECULAR DEVICES (USA). The range of reference values and the intra-and inter-assay variation coefficient of plasma CA-125 provided by the manufacturer were up to 35 U/mL, 1.3%, and 5.5%, respectively. However, for dialysate there is no cut-off point or reference values. The range of reference values and the coefficient of intra and inter-assay variation of plasma CCL2 provided by the manufacturer were 134 to 436 pg/mL, 5.8%, and 5.7%, respectively.

### Covariables

The covariables of the study were sex, age, time in months of PD, body mass index (BMI), systolic and diastolic blood pressure (mmHg), presence of diabetes mellitus (DM), primary cause of CKD, and medication use.

### Statistical analysis

Categorical variables were presented using proportions and continuous variables using means and standard deviation or medians and interquartile range. Correlations between CCL2 plasma and dialysate levels, and the correlation between the explanatory variable (CCL2 plasma and dialysate levels) with the response variable (CA-125 levels) were investigated through Spearman’s correlation (rho), since they presented an asymmetric distribution, observed by the analysis of histograms. The magnitude of the correlation was classified into: rho values up to 0.40 - weak correlation; rho 0.41 to 0.70 - moderate correlation; and rho above 0.70 - strong correlation 19.

To investigate whether plasma and CCL2 dialysate levels and the response variable (CA-125 levels in dialysate) were independently associated, crude ratio of arithmetic means (RoM) ratios were estimated and adjusted using generalized linear models (family Range and logarithmic link function). This model does not consider the assumption of normality in the distribution of response variables. After univariate analysis, the RoM were adjusted for the main variables that could confuse the association found, according to the literature 11 ^,^ 14 ^,^ 20 and to data collected by the study. The crude RoMs were adjusted for age (Model 1) then for time on PD (Model 2). Associations with p value <0.05 were considered statistically significant. All other covariables described in this article were maintained only in the descriptive analysis of the data. All analyzes were performed using the statistical program STATA, version 14.0.

### Ethical aspects

This study was approved by the Research Ethics Committees of the Federal University of São João Del-rei and the São João de Deus Health Complex - CAAE - 19284613.5.0000.5545 and all participants signed the informed consent form.

## Results

### Clinical characteristics of the population

Most participants were male (51.2%), with a mean age of 63.1 years, and a median time in PD of 27 months (IQ = 14-42). On average, they had a BMI of 24.5 (SD = 4.4), 58.5% had DM and had an average systolic blood pressure of 142.0 (SD = 20.9) mmHg and median diastolic blood pressure of 80 (IQ = 80-90) mmHg. The most prevalent primary disease was diabetic nephropathy (31.7%), followed by hypertensive nephrosclerosis (24.4%). The most used antihypertensives were diuretics (85.4%), followed by angiotensin receptor antagonists (ARA) (53.7%) and the (-blockers (48.4%). In addition, 31.7% of participants used insulin and 56.1% used statins. The median dialysate concentration of CA-125 was 17 U/mL and of CCL2 was 278.4 pg/mL (Table 1).

**Table 1:** Distribution of sociodemographic and clinical characteristics of PD patients. Divinópolis - Minas Gerais - Brazil

Variables	n=41
Ages (years)	63.1 (14.9)
Gender	
Male [n (%)]	21 (51.2%)
Primary causes of CKD [n (%)]	
Diabetic nephropathy	13 (31.7%)
Hypertensive nephrosclerosis	10 (24.4%)
CGN	8 (19.5%)
PKD, CAKUT and obstructive uropathy	6 (14.6%)
Unknown etiologies	4 (9.8%)
Blood pressure	
Systolic pressure (mmHg)	142.0 (20.9)
Diastolic pressure (mmHg)	80 (80-90)
Diabetes	24 (58.5%)
BMI (kg/m^2^)	24.5 (4.4)
Use of medicines	
β-blockers	22 (48.4%)
Calcium channel antagonists	17 (35.5%)
ARA	22 (53.7%)
ACEI	2 (4.9%)
Diuretics	35 (85.4%)
Anxiolytics/Antidepressants	16 (39.0%)
Vitamin Supplements	15 (32.2%)
Acetylsalicylic acid	20 (48.8%)
Statins	23 (56.1%)
Insulin	13 (31.7%)
Time on PD (months)	27 (14-42)
CA-125 (U/mL)	17 (8.7–28.7)
CCL2 (pg/mL)	278.40 (114.6-448)

The results are presented as mean and standard deviation for data with symmetric distribution, median (interquartile range) for data with the asymmetric distribution. Categorical variables are presented using proportions: n (%). BMI: body mass index; CKD: chronic kidney disease; CNG: Chronic Glomerulonephritis; PKD: Polycystic Kidney Disease; CAKUT: Congenital anomalies of the kidneys and urinary tract; PD: peritoneal dialysis; ARA: angiotensin receptor antagonists; ACEI: Angiotensin-Converting Enzyme Inhibitors.

### Plasma measurements

**Figure 1:**
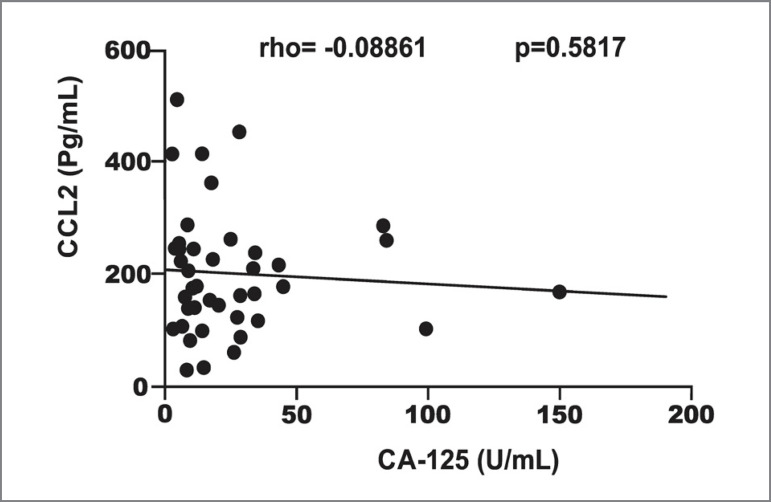
Correlation of CA-125 dialysate levels with the plasma levels of CCL2.

When investigating the correlation between plasma and dialysate levels of CCL2, no statistically significant correlation was found (rho=0.04, p-value=0.79). There was also no statistically significant correlation between the plasma levels of CCL2 and the levels of CA-125 in the dialysate (Figure 1).

No statistically significant association was found between the plasma levels of CCL2 and the levels of CA-125 in the dialysate, neither in univariate analysis nor after adjustment for confounding variables (Table 2).

**Table 2:** Crude and adjusted ratios of arithmetic means (RoM) of the association between CA-125 levels in the dialysate and plasma CCL2 levels. Divinópolis - Minas Gerais - Brazil

	Multivariate		
**CCL2**	**Crude**	**Model 1**	**Model 2**
	**RoM (IC95%)**	**RoM (IC95%)**	**RoM (IC95%)**
	0.96 (0.80-1.16)	0.96 (0.81-1.16)	0.96 (0.81-1.17)

The RoM obtained by the exponential of the parameter resulting from the Generalized Linear Model with Gamma distribution. CA-125: Cancer Antigen - 125; CCL2: C-C motif chemokine ligand 2

Model 1: RoM crude + age.

Model 2: Model 1+ time on PD (months)

### Dialysate measurements

**Figure 2:**
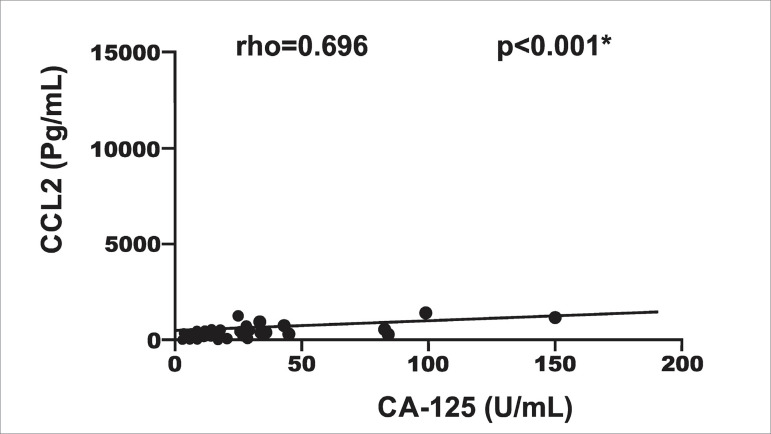
Correlation between CA-125 levels and CCL2 levels in the dialysate.

A significant, moderate and positive correlation was observed between the levels of CA-125 and CCL2 in the dialysate (rho = 0.696; p <0.05) (Figure 2).

A significant association was found between the levels in the CCL2 dialysate and the levels of CA-125 (RoM=1.31; CI=1.20-1.43; p<0.05) in the univariate analysis. For each increased unit of measurement in the levels of CCL2 in the dialysate, the levels of CA-125 in the dialysate were, on average, 31% higher. This association remained statistically significant after adjusting for age (RoM=1.31; IC=1.19-1.44; p<0.05) and for time in months of PD (RoM=1.34, IC=1.22-1.48; p<0.05). For each increased unit of measurement in the levels of CCL2 in the dialysate, the levels of CA-125 in the dialysate were, on average, 34% higher, even after adjustments for all confounding variables (Table 3).

**Table 3:** Crude and adjusted ratio of arithmetic means (RoM) of the association between levels of CA-125 and CCL2 in the dialysate of patients with CKD on PD. Divinópolis - Minas Gerais - Brazil

	Multivariate		
**CCL2**	**Crude**	**Model 1**	**Model 2**
	**RoM (IC95%)**	**RoM (IC95%)**	**RoM (IC95%)**
	1.31 (1.20-1.43)*	1.31 (1.19-1.44)*	1.34 (1.22-1.48)*

The RoM obtained by the exponential of the parameter resulting from the Generalized Linear Model with Gamma distribution. CA-125: Cancer Antigen - 125; CCL2: C-C motif chemokine ligand 2

Model 1: RoM crude + age.

Model 2: Model 1+ time on PD (months)

## Discussion

This study involving patients on peritoneal dialysis showed that increased levels of CA-125 in the dialysate are associated with increased levels of CCL2 in the dialysate, even after adjustments for important confounding factors, such as age and time on PD, evaluated in the multivariate model. Also, no statistically significant correlation was found between the plasma and dialysate levels of CCL2, just as there was no statistically significant correlation or association between the CCL2 plasma levels and the CA-125 dialysate levels.

These findings corroborate the hypothesis that the increase in CCL2 in dialysate due to local inflammation of peritoneal membrane causes damage to the mesothelial cells of the peritoneum and, consequently, increases the CA-125 levels. They also reinforce that the chronic systemic inflammation, characteristic of the dialysis process, may not influence the high levels of these biomarkers in dialysis, since we did not observe an association between serum and dialysate levels. Lambie et al. (2013) also reported that CCL2 present in dialysate is produced predominantly within the peritoneum in patients on PD. Other studies show that the CA-125 present in the dialysate is predominantly produced inside the peritoneum, with minimal influence to serum levels, mostly because of the molecular weight that makes it impossible to pass through the pores of the peritoneal membrane 21 ^,^ 22.

CCL2 has been suggested as an important chemoattractant of monocytes and macrophages to the peritoneal region, being the main cells present in inflammatory processes, especially in peritonitis episodes. Other studies have also shown that CCL2 is involved in the process of neoangiogenesis in peritoneal membrane 14 ^,^ 23. With the increase of new blood vessels in peritoneal membrane, the patient in PD would have an increase in the rate of solute transport, which would result in a decrease in ultrafiltration, culminating in the loss of the dialysis capacity of the peritoneal membrane. Besides, CCL2 participates in the initiation and progression of peritoneal fibrosis, which can modify the biology of resident cells, stimulating epithelial-mesenchymal transition of the peritoneal membrane, and once again promoting the loss of its functionality 24.

Studies have shown that during the process of peritoneal membrane degradation, CA-125 levels are increased in the peritoneal cavity, and tend to decrease as peritoneal membrane fibrosis occurs; therefore, it can be inferred that levels of CA-125 in the dialysate may be an important biomarker of inflammation and of the integrity of the mesothelial mass of the peritoneum 12.

Changes in the concentrations of CA-125 in the dialysate vary over time regardless of the occurrence of infectious processes characteristic of PD (peritonitis). This fact probably indicates changes in the cellular profile of peritoneal membrane in patients undergoing PD^25^. However, due to the great inter-individual variability, possibly caused by differences in the number of mesothelial cells that express CA-125, a single measurement is generally not decisive and informative for decision making.

Therefore, longitudinal studies are recommended to evaluate the levels of CA-125 in dialysate over the duration of PD, not only as a marker of the integrity of the peritoneal membrane, but also as a possible biomarker of the local inflammatory process. A rise in the levels of CA-125 in the dialysate suggest a greater damage of mesothelial cells of the peritoneal membrane, supposedly due to a local inflammatory process. It is believed that the main importance of identifying CA-125 levels in the dialysis of PD patients to determine loss of peritoneal membrane function is the mesothelial cells quantification and individualized follow-up. This information would allow to observe the increase in the levels of this molecule in the short term, revealing a local inflammatory process, loss of mesothelial cells, and a consequent increase in the transport rate of solutes by the peritoneal membrane, which, depending on the individual’s residual diuresis, may contraindicate the permanence in DP. In this context, the levels of CA-125 could indicate an earlier exit of the patient from this type of dialysis, without necessarily having to wait for the deleterious effects of inadequate dialysis.

The results of this study can be used in clinical practice for decision making and patient maintenance in PD based on concrete data on its viability. However, it is important to note some limitations. Among them, the cross-sectional study design does not allow making causal inference, since this design does not guarantee temporality. In addition, we used some information from a secondary source (patient records) that are not always clear. Moreover, the sample size of our study was small, despite providing data of 74 PD patients from a major center of nephrology in Brazil.

As for strengths, this was the first study carried out in Brazil that suggests the use of CA-125 as a possible inflammatory biomarker and the use of a multivariate analysis model that guarantees greater robustness to the investigation.

In conclusion, our findings show that levels of CCL2 in the dialysate are associated with levels of CA-125 also in the dialysate in PD patients, even after adjustment for important confounding factors. This finding indicates the role of CA-125 in the local inflammatory process, in addition to reinforcing the action of CCL2 in inflammation in PD patients. The association of CA-125 with CCL2 may indicate that the local inflammatory process leads to temporary or definitive changes (fibrosis) of the peritoneal membrane, and, consequently, the loss of the integrity of the peritoneal membrane and its failure in the ultrafiltration process. A better understanding of this pathogenesis could contribute to the discovery of new inflammatory biomarkers, but more studies still need to be carried out, especially longitudinal ones, to assess whether these biomarkers could be useful for decision-making in the management of PD patients, aiming to prolong the useful life of peritoneal membranes, as well as the PD technique.
